# Fetal gender and pregnancy outcomes in Libya: a retrospective study

**DOI:** 10.3402/ljm.v8i0.20008

**Published:** 2013-01-08

**Authors:** Mounir M. Khalil, Esgair Alzahra

**Affiliations:** Al-Jalaa Maternity Hospital, Tripoli, Libya

**Keywords:** fetal gender, fetal sex, pregnancy outcomes, cesarean rates, pregnancy risk factors

## Abstract

**Objective:**

The relationship between pregnancy outcomes and fetal gender is well reported from different areas in the world, but not from Africa. In this study, we try to understand whether the recorded phenomenon of association of adverse pregnancy outcomes with a male fetus applies to our population.

**Materials and methods:**

A total of 29,140 patient records from 2009 and 2010 were retrieved from Aljalaa Maternity Hospital, Tripoli, Libya. Analysis was carried out to find the correlation between fetal gender and different pregnancy outcomes.

**Results:**

A male fetus was associated with an increased incidence of gestational diabetes mellitus (odds risk 1.4), preterm delivery (6.7% for males, 5.5% for females, odds risk 1.24), cesarean section (23.9% for males, 20% for females, odds risk 1.25), and instrumental vaginal delivery (4.4% for males, 3.1% for females, odds risk 1.48), *p*<0.005. Preeclampsia was more frequent among preterm females and postterm males, *p*<0.005. It was also more frequent in male-bearing primigravids, *p*<0.01.

**Conclusion:**

We confirm the existence of an adverse effect of a male fetus on pregnancy and labor in our population. We recommend further research to understand the mechanisms and clinical implications of this phenomenon.

Since Hall and Carr-Hill (1) first described the relationship between fetal gender and pregnancy outcomes 30 years ago, several studies investigating this phenomenon in different parts of the world have been published. Most work was carried out in western countries (1–7). Significant evidence points toward an adverse effect of a male fetus on the outcome of pregnancy in terms of higher rates of gestational diabetes mellitus (GDM) (4), preeclampsia (PET) (8), eclampsia (9), fetal macrosomia, failure to progress during the first and second stages of labor, cord prolapse, nuchal cord, true umbilical cord knots (7), preterm birth, and premature preterm rupture of membranes (4, 10). Cesarean sections (2–7, 11) and instrumental deliveries (7, 11) were also more frequently found among male neonates compared with females. Meanwhile, only theories have been proposed to explain these findings without providing a full understanding for the etiology.

Among these studies, no data were published from Africa. To investigate the presence of this phenomenon in our population, we studied the association between fetal gender and pregnancy outcome in Al-Jalaa Maternity Hospital in Tripoli, Libya. This hospital is one of three obstetrics and gynecology tertiary centers in the Libyan capital, Tripoli. It has an annual rate of approximately 16,000 deliveries.

## Methods and materials

Data were retrieved retrospectively from the statistics department in Al-Jalaa Maternity Hospital for all patients presented with diagnosis of ‘labor’ or ‘delivery’ and admitted to the labor ward (this also includes those who presented immediately after home/car delivery) from January 1 2009 to December 31 2010.

Inclusion criteria were singleton pregnancies that completed 28 weeks of gestation (by last menstrual period or early scan). Stillbirths, neonatal deaths, and infants with congenital anomalies were excluded. The Aljalaa Hospital authorities approved the study.

Data collected from patient records (*n*=29,051) included maternal age, parity, gestational diseases (preeclampsia, GDM), gestational age on date of delivery, mode of delivery, neonatal weight, and gender. Records with missing data (911, 3.24%) were excluded from the study, leaving 28,140 complete records for the study.

Points studied included the effect of fetal gender on the incidence of maternal gestational disease (namely GDM and preeclampsia), risk of preterm delivery, and the need for operative intervention during delivery.

Statistics were performed using the SPSS statistics software version 17.0. Data were presented as mean ± SD. Pearson chi-square test of independence was used for correlating maternal disease, term status, and modes of delivery with fetal gender. An independent sample *t*-test was used with gestational age and birth weight. Correlation with control variables was done using partial correlations. Odds risk, representing sex ratio, was also calculated where appropriate. *p* values were considered significant at values <0.05.

## Results

From the selected 28,140 deliveries during the 2-year period (2009 and 2010), 14,491 (51.5%) had male neonates, 13,649 (48.5%) had females; 10,045 (35.7%) were primigravid, 16,377 (58.2%) were multiparous while 1,718 (6.1%) were grand multiparous (parity of 5 or more). Mean maternal age was 30.87 ± 5.66 years. Other data of pregnancies are listed in Table 1.

**Table 1:** Data of study population

	Males *n*=14,491 (51.5%)	Females *n*=13,649 (48.5%)	*p*	M:F ratio
GDM	242 (1.7%)	163 (1.2%)	<0.001	1.4
Preeclampsi	452 (3.1%)	410 (3%)	0.58	–
Gestational age GA				
Preterm	976 (6.7%)	754 (5.5%)	<0.005	1.24
Term	12,959 (89.4%)	12,347 (90.5%)	–	–
Postterm	556 (3.8%)	548 (4%)	<0.005	–
Mean GA (weeks)	39.12 ± 1.77	39.23 ± 1.79	<0.001	–
MOD				
Vaginal delivery	10,645 (73.5%)	10,281 (75.3%)	–	–
Non-instrumental	10,438 (98.1%)	10,137 (98.6%)	–	–
Instrumental	207 (1.9%)	144 (1.4%)	<0.005	1.4
Cesarean section	3,846 (26.5%)	3,368 (24.7%)	<0.001	1.1
Elective	1,362 (35.4%)	1,268 (37.6%)	–	–
Emergency	2,484 (64.6%)	2,100 (62.4%)	–	–
Mean birth weight	3,400 ± 572 g	3,296 ± 553 g	<0.001	–

M, male; F, female; GA, gestational age; MOD, mode of delivery.

GDM was significantly higher among male-bearing women than female-bearing women, with a male to female sex ratio of 1.4 (95% CI: 1.15–1.7, *p*<0.001). This association also persisted after controlling for maternal age (*p*<0.001). Male birth weights (4,026 ± 835 g) were higher than female birth weights (3,597 ± 734 g) among women with GDM, with a mean difference of 428 g (*p*<0.001).

The overall incidence of preeclampsia (PET) showed no significant relation to fetal gender, *p*=0.58. However, there is a bimodal distribution of PET among both genders at different gestational ages. PET was significantly higher in female-bearing pregnancies with preterm labor (9.5% for females: 5.1% for males, *p*<0.005), while it was significantly higher in male-bearing pregnancies for postterm delivery (4% in males, 1.1% in females, *p*<0.005, Table 2).

**Table 2:** Preeclampsia in primigravids at different gestational ages

Gestational age	Males 425:52.4%	Females 410:47.6%	*p*	Sex ratio
Preterm	50 (5.1%)	72 (9.5%)	<0.005	F:M is 1.4
Term	380 (2.9%)	332 (2.7%)	0.24	–
Postterm	22 (4.0%)	6 (1.1%)	<0.005	M:F is 2.35

M, male; F, female.

PET also increased among male-bearing primigravid pregnancies compared to female-bearing primigravids, with a male to female ratio of 1.17 (95% CI: 1.04–1.32, *p*<0.01).

The mean gestational age for male neonates (39.1±1.8 weeks) was significantly lower than for females (39.2±1.8 weeks, *p*<0.001). This resulted in a significantly higher rate of preterm deliveries among males (OR=1.24, 95% CI: 1.1–1.4, *p*<0.005). This difference remained significant after controlling for neonatal birth weight *p*<0.005.

Mode of deliveries were analyzed in the total population. Male babies had a higher chance of delivery by cesarean section than female babies (26.5% *vs* 24.7%, OR=1.10, 95% CI: 1.05–1.16, *p*<0.001). For vaginal deliveries, male babies also had a higher rate of operative intervention (vacuum and forceps) (1.9% *vs* 1.4% of vaginal deliveries, OR=1.4, 95% CI: 1.1–1.7, *p*<0.005).

For the subgroup of primigravid delivered at term without planned cesarean section (Table 3), male babies were significantly associated with cesarean delivery relative to female babies (23.9%, 20%, OR=1.25, 95% CI: 1.13–1.39, *p*<0.005).

**Table 3:** Data for primigravid term patients not planned for cesarean section

	Males 4,400:51.3%	Females 4,171:48.7%	*p*	M:F ratio
MOD				
Vaginal delivery	3,350 (76.1%)	3,335 (80%)	–	–
Non-instrumental	3,201 (95.6%)	3,233 (96.9%)	–	–
Instrumental	149 (4.4%)	102 (3.1%)	<0.001	1.48
Emergency CS	1,050 (23.9%)	836 (20%)	<0.005	1.25
Mean birth weight	3,301 ± 500 g	3,205 ± 477 g	<0.001	–

M, male; F, female; GA, gestational age; CS, cesarean section; MOD, mode of delivery.

A significant association of gender with instrumental delivery was also observed *p*<0.001 (4.4% of male vaginal deliveries *vs* 3.1% of female vaginal deliveries, OR=1.48, 95% CI: 1.14–1.9). All of these associations persisted despite controlling for birth weight, which was higher in males (3,301 ± 500 g) than females (3,205 ± 477 g, *p*<0.001).

## Discussion

In the literature, observations about the adverse effect of a male fetus on pregnancy and labor have been reported in many societies (1–8). Although this association is statistically well established, there is neither an accepted explanation nor a clear evidence of its clinical relevance.

Our study, conducted in Libya, a developing country in North Africa, showed the same association of male fetus with increased GDM, PET at advanced gestational age, preterm labor, higher birth weight, and increased cesarean and operative deliveries.

The relationship between fetal gender, birth weight, and GDM is a complicated issue with some debate in the literature (12, 13). Male gender is associated with increased rates of GDM and increased birth weights. In turn, GDM results in higher birth weight. Our results showed a greater difference between male birth weights and female birth weights in women with GDM (mean difference in normal population 104 g *vs* 428 g in GDM women). We also found a general association between male-bearing and developing GDM, even after controlling for maternal age, another risk factor for the disease. Further investigations like stage of the disease and gestational age of onset are beyond the scope of this study.

PET prevalence in preterm females and postterm males was previously reported (8) and a relationship to differences in implantation among sexes was suggested as an explanation for such distribution (8). PET association with male-bearing primigravids was also reported in the literature (14).

In general, males tended to be born before females. At lower gestational ages, more males were born than females. Preterm deliveries occurred more frequently among males (6.7%) than females (5.5%) *p*<0.001, while postterm deliveries were more frequent among females (4%) than males (3.8%) *p*<0.005.

Although it is known that preterm delivery is associated with low rather than high fetal weight (15), the difference was still significant after we controlled the gender ratio in preterm deliveries for birth weight.

There is special interest in the association of male-bearing with increased rates of cesarean deliveries. Although some concluded that this was due to higher male neonatal birth weights resulting in difficult labor (11, 12), studies that controlled for this cofactor still had significantly higher cesarean deliveries in male deliveries. (6, 16)

In our study, after finding significant associations between operative deliveries (cesarean sections and instrumental deliveries) and male babies, we restudied the primigravid women with term deliveries that were not planned for elective cesarean separately. After adjustment for birth weight, the results still showed a significant association between male gender and cesarean section.

Our results, added to the published material, suggest that this phenomenon is not dependent on race, ethnicity, or environmental conditions, and seems to be biological in origin.

Until now, the physiological bases of these phenomena remain obscure (11), and work on the etiology is insufficient. The adverse effect of a male fetus has been reported even in heterosexual twins (17). Nevertheless, few theories were introduced relating these differences to different patterns of fetal development among sexes, higher male fetus metabolic rate, endocrine factors, immunological reactions, longer male umbilical cord (with its sequels), and higher birth weight of males (2).

The well-established statistical data should not be translated into any kind of clinical guideline until further research finds if gender-based decisions would improve the outcomes or not.

The study's drawback is the limitation of available details. Some useful information was missing, such as abortion rates, hyperemesis and other gestational complications, labor progress details, indications for operative interventions, and neonatal biometry (head and abdominal circumferences). Apgar score, early neonatal death (and hence perinatal mortality rate), and admission to nursery details were also unavailable.

## Conclusion

In the Libyan population, we confidently report that a male fetus is an independent adverse factor for pregnancy outcome, that is, maternal gestational diabetes, post-term preeclampsia, preeclampsia in primigravids, preterm deliveries, cesarean sections, and instrumental delivery. Further work is recommended to understand the relevance of this knowledge to clinical practice.
